# Video calls did not reduce PTSD symptoms in relatives during restricted ICU visits in the COVID-19 pandemic

**DOI:** 10.1038/s41598-022-18616-8

**Published:** 2022-08-24

**Authors:** Bjoern Zante, Katja Erne, Marie-Madlen Jeitziner

**Affiliations:** 1 Department of Intensive Care Medicine, Inselspital, Bern University Hospital, University of Bern, Freiburgstrasse 10, 3010 Bern, Switzerland; 2 Institute of Nursing Science (INS), Department of Public Health (DPH), Faculty of Medicine, University of Basel, Basel, Switzerland

**Keywords:** Psychology, Quality of life

## Abstract

To help reduce the spread of the SARS-CoV-2 virus during the COVID-19 pandemic, ICU visits were banned or restricted. Therefore, family-centered care as usually practiced was not feasible Video calls were recommended to meet relatives’ needs. The aim of this study was to investigate the effect of video calls on symptoms of post-traumatic stress disorder (PTSD) in relatives of ICU patients. This single-center study was performed during the first wave (15.03.2020‒30.04.2020; visits banned) and the second wave (01.10.20‒08.02.21: visits restricted) of the COVID-19 pandemic. The Impact of Event Scale-Revised (IES-R) was used to assess PTSD symptoms and an adapted version of the Family Satisfaction in the Intensive Care Unit 24-Item-Revised questionnaire (aFS-ICU 24R) to assess family satisfaction 3 months after ICU stay. The primary outcome was the difference in IES-R score at 3 months between the video call group (VCG) and the standard care group (SCG, no video calls). In addition, inductive content analysis of relatives’ comments regarding their satisfaction with decision-making and ICU care was performed. Fifty-two relatives (VCG: n = 26, SCG: n = 26) were included in this study. No significant difference in IES-R scores was observed between the VCG and the SCG (49.52 ± 13.41 vs. 47.46 ± 10.43, *p* = 0.54). During the ICU stay (mean 12 days, range 5.25‒18.75 days), the members of the VCG made a median of 3 (IQR 1‒10.75) video calls. No difference between the groups was found for conventional telephone calls during the same period (VCG: 9 calls, IQR 3.75‒18.1; SCG: 5 calls, IQR 3‒9; *p* = 0.12). The aFS-ICU 24R scores were high for both groups: 38 (IQR 37‒40) in the VCG and 40 (IQR 37‒40: *p* = 0.24) in the SCG. Video calls appeared largely ineffective in reducing PTSD symptoms or improving satisfaction among relatives affected by banning/restriction of ICU visits during the COVID-19 pandemic. Further investigations are needed to acquire more data on the factors involved in PTSD symptoms experienced by relatives of ICU patients during the COVID-19 pandemic.

## Background

In 2020 the onset of the COVID-19 pandemic disrupted the long-established practice of family-centered care in intensive care units (ICU)^1,2^. ICU visits were either restricted or banned altogether to reduce the spread of the SARS-CoV-2 virus. The normal integration of relatives, family conferences for shared decision-making, direct caregiving, and frequent face-to-face communication were no longer feasible^3–6^. This resulted in an extraordinary burden on relatives of patients in the ICU^3,4^. The prevalence of post-traumatic stress disorder (PTSD) symptoms has been found to range up to 69%^7^ and appears higher than before the pandemic (33‒56%)^8–11^. Prior to the pandemic, only a small number of interventions were effective in reducing anxiety, depression, or PTSD in relatives^12^. New approaches have been proposed to meet the needs of patients’ relatives during restricted ICU visiting in the pandemic^5,12^. For instance, video calls have been recommended for maintaining communication between relatives and ICU staff^13–16^. However, data on the effect of video calls on PTSD symptoms and family satisfaction are sparse.

Therefore, we conducted a prospective mixed-method study to investigate the effect of video calls versus standard care on the occurrence of PTSD symptoms in relatives of ICU patients.

## Methods

### Study design

This prospective single-center mixed-method study was performed in a multidisciplinary department of intensive care medicine at a tertiary academic medical center (Inselspital, University Hospital Bern, Switzerland) between March 2020 and February 2021.

Relatives were interviewed by telephone, using the Impact of Event Scale-Revised (IES-R) to assess PTSD symptoms 3 months after ICU stay. Furthermore, we performed inductive content analysis on additional comments made by family members regarding their satisfaction with decision making and ICU care in general.

To help prevent the spread of the SARS-CoV-2 virus, ICU visits were suspended (except for end-of-life visits) during the first wave of the pandemic (15.03.20‒30.04.20) and restricted (to one relative per day for 1 h) during the second wave (01.10.20‒08.02.21). Most of the video calls were made during the first wave of the COVID-19 pandemic, when only end-of-life visits to the ICU were permitted.

### Participants

All relatives of patients hospitalized in the ICU during the observation period were potentially eligible for the study. The exclusion criteria were insufficient knowledge of German, French, or Italian, unwillingness to participate in the study, and age < 18 years.

We adopted a convenience sampling strategy^17^ to ensure that all relatives who were interested in the video call service were able to use it (as part of the video call group, VCG) according to published recommendations. Relatives without the technical equipment for video calls (e.g., cell phone, tablet, or internet access) were assigned to the standard care group (SCG). Only one relative per patient participated in the study. Critical care nurses identified relatives potentially eligible for video calls during telephone calls. Specially assigned nurses conducted the video calls with the relatives by appointment. The frequency of the video calls was agreed individually with each relative.

### Ethics

The Ethics Committee on Human Research of the canton of Bern (*Kantonale Ethikkommission*) waived the requirement for ethics approval and the need to obtain consent for the collection, analysis, and publication of the recorded data (KEK Req-2020-00404). The study adhered to the tenets of the Declaration of Helsinki.

### Outcome measures

The IES-R was used to assess PTSD symptoms in the participating relatives^18–21^. This validated questionnaire contains 22 items comprising three defined subscales (avoidance, intrusion, and hyperarousal), with ratings on a five-point Likert scale from 0 (“not at all”) to 4 (“extremely”). A score from 0 (best) to 88 (worst) could be achieved. An IES-R score of 33 was used as cut-off for severe PTSD symptoms, as in previously published studies^7,20,22^.

The Family Satisfaction in the Intensive Care unit 24-Item-Revised (FS-ICU 24R) was used to assess family satisfaction during the ICU stay^7,23^. The questionnaire was adapted and validated to allow for the special conditions of the suspended/restricted ICU visiting policy (aFS-ICU 24R)^7^. In brief, questions assuming physical presence in the ICU were excluded. Eight of nine items were rated on a five-point Likert scale from 1 (“very dissatisfied”) to 5 (“completely satisfied”) and one from 1 (“I felt very excluded”) to 5 (”I felt very included”), yielding a total score between 9 (worst) and 45 (best). Three months after each patient’s discharge from or death in the ICU, the relatives were interviewed by telephone using the IES-R and the aFS-ICU 24R. Furthermore, the relatives’ comments and experiences were recorded for qualitative inductive content analysis.

The data recorded on the relatives included demographics (age, gender, relationship to the patient, previous ICU experience). Furthermore, family-centered care items such as keeping of an ICU diary (yes/no), care team involved (yes/no), number of telephone calls (by study participant and total calls by all relatives), and number of video calls with the participating relative were recorded.

The data recorded on the patients included demographics (age, gender), emergency admission (yes/no), COVID-19 positivity (yes/no), mechanical ventilation (yes/no), duration of mechanical ventilation (hours), length of stay in ICU (days), Acute Physiology and Chronic Health Evaluation II (APACHE II) score, Simplified Acute Physiology Score II (SAPS II), and death in the ICU (yes/no).

The primary outcome of this study was the difference in IES-R score at 3 months between the VCG and the SCG. Secondary outcomes were the differences in the relatives’ and patients’ indices regarding IES-R < 33 (absence of severe PTSD- related symptoms) vs. IES-R ≥ 33 (presence of severe PTSD-related symptoms) after 3 months and comparisons between the first and second waves of the pandemic^20,24^.

### Qualitative data

Based on the open questions in the aFS-ICU 24R, relatives’ comments on their satisfaction with decision making and with care in general were included in inductive content^23,25^. The family comments were recorded by hand during the telephone survey (by MMJ and KE). These data were used for hypothesis generation regarding potential factors contributing to PTSD symptoms. The analysis was conducted by two experienced researchers (MMJ, KE) until agreement was reached on relevant categories.

### Statistical analysis

Continuous indices were computed as mean and standard deviation (SD) or median and interquartile range (IQR), categorical indices as proportions. The Shapiro‒Wilk test was used to check normal distribution. Comparisons of continuous indices between groups [defined by video calls (yes/no), IES-R cut-off, and wave (first/second)] were compared using the Mann‒Whitney U-test or the t-test, as appropriate. Categorical indices were compared using the chi-square test or Fisher’s exact test, as appropriate. Statistical analysis was performed using MedCalc® Statistical Software version 19.6 (MedCalc Software Ltd, Ostend, Belgium).

A power analysis was performed for sample size estimation. We assumed a mean IES-R score of 50 in the SCG and hypothesized a 10-point reduction in IES-R score for the VCG. We calculated that a total of 52 relatives would be needed to have 80% power to detect this difference with a two-sided alpha level of 0.05.

## Results

### Study population

A total of 52 relatives, each with one family member in the ICU, were included in the study (VCG: n = 26, SCG: n = 26). Their demographic data are given in Table 1.

**Table 1:** Characteristics of relatives and patients.

Characteristics	*N*	All participants	Video call group	Standard care group	*p* value
**Relatives**					
Age, years	45	48.78 ± 14.78	47.86 ± 15.81	49.65 ± 13.81	0.53
Female gender, % (no.)	52	73.1 (38)	80.8 (21)	65.4 (17)	0.22
IES-R score at 3 months	52	48.29 ± 11.97	49.12 ± 13.5	47.46 ± 10.43	0.2
IES-R > 33, % (no.)	52	88.5 (46)	84.6 (22)	92.3 (24)	0.38
aFS-ICU 24R at 3 months	52	40 (37‒40)	37 (37–40)	40 (37‒40)	0.27
Family member in ICU	52				
Spouse, % (no.)		36.5 (19)	38.5 (10)	34.6 (9)	0.78
Parent, % (no.)		9.6 (5)	7.7 (2)	11.5 (3)	0.67
Sibling, % (no.)		7.7 (4)	3.8 (1)	11.5 (3)	0.61
Child, % (no.)		44.2 (23)	46.2 (12)	42.3 (11)	0.78
Other, % (no.)		1.9 (1)	3.8 (1)	0 (0)	1
ICU diary, % (no.)	50	42 (21)	50 (12)	34.6 (9)	0.28
Previous ICU experience, % (no.)	52	42.3 (22)	46.2 (12)	38.5 (10)	0.58
Care team involved, % (no.)	50	18 (9)	16.7 (4)	19.2 (5)	1
Telephone calls with nurses, no	50	6 (3–15)	8.5 (3.5–18.5)	5 (3–9)	0.14
Video calls, no	52	0.5 (0–3.5)	**3.5 (1–11)**	**0 (0)**	**< 0.0001**
ICU visits, no	50	1 (0‒3)	**0 (0–1.5)**	**3 (1‒5)**	**0.0007**
**Patients**					
Age, years	52	59.8 ± 15.27	59.73 ± 14.1	59.89 ± 16.66	0.97
Female gender, % (no.)	52	59.6 (31)	**23.1 (6)**	**57.7 (15)**	**0.01**
Emergency admission, % (no.)	52	96.2 (50)	92.3 (24)	100 (26)	0.49
APACHE II	44	27.45 ± 8.23	**28.14 ± 6.06**	**26.77 ± 10.05**	**0.03**
SAPS II	50	37.8 ± 19.36	41.21 ± 21.62	34.52 ± 16.69	0.23
COVID-19, % (no.)	52	38.5 (20)	**53.8 (14)**	**23.1 (6)**	**0.02**
Mechanical ventilation, % (no.)	52	88.5 (46)	96.2 (25)	80.8 (21)	0.09
Duration of mechanical ventilation, hours	47	115.8 (46.5–293.3)	114.1 (54.6–298.2)	121.8 (35.7–304.33)	0.86
ICU length of stay, days	52	8 (5–18)	10 (5–19)	6 (4–13)	0.11
Died in ICU, % (no.)	52	9.6 (5)	11.5 (3)	7.7 (2)	1

Significant values are in bold.

Age, IES-R, Acute Physiology and Chronic Health Evaluation II (APACHE II) score, and Simplified Acute Physiology Score II (SAPS II) expressed as mean and standard deviation; aFS-ICU 24R, telephone calls, ICU visits, duration of mechanical ventilation, and length of stay expressed as median and interquartile range.

### Comparison of characteristics related to video calls

Detailed data are given in Table 1. No significant differences were found between the VCG and the SCG in IES-R and aFS-ICU 24R scores. The numbers of ICU visits by the participating relative and total ICU visits by all of the patient’s relatives were higher in the SCG. Intergroup differences in the characteristics of the patients were observed for gender (more females in the SCG), APACHE II score, and COVID-19 positivity (both higher in the VCG). In the VCG, 16 relatives participated during the first wave and 11 during the second wave of the pandemic. All of the relatives in the SCG participated during the second wave.

### Comparison of characteristics related to IES-R

No significant differences in relatives’ and patients’ characteristics were observed between the IES-R subgroups (< 33 versus ≥ 33), except for the IES-R score itself (Table 2).

**Table 2:** Comparison of characteristics related to IES-R score at 3 months after ICU stay.

Characteristic	IES-R < 33	IES-R ≥ 33	*p* value
**Relatives**			
Age, years	55.6 ± 20.28	47.92 ± 13.93	0.28
Female gender, % (no.)	66.7 (4)	73.9 (34)	0.66
**IES-R score at 3 months**	**26.83 ± 1.94**	**51.09 ± 9.63**	**< 0.0001**
aFS-ICU 24R at 3 months	40 (37‒40)	40 (37‒44)	0.56
Family member in ICU			
Spouse, % (no.)	50 (3)	34.8 (16)	0.66
Parent, % (no.)	0 (0)	10.9 (5)	1
Sibling, % (no.)	0 (0)	8.7 (4)	1
Child, % (no.)	33.3 (2)	45.7 (21)	0.68
Other, % (no.)	16.7 (1)	0 (0)	0.12
ICU diary, % (no.)	83.3 (5)	36.4 (16)	0.07
Previous ICU experience, % (no.)	50 (3)	41.3 (19)	0.69
Care team involved, % (no.)	0 (0)	20.5 (9)	0.58
Telephone calls with nurses, no	3.5 (1‒5)	7 (3‒16)	0.25
Video calls, no	1 (0‒13)	0 (0‒3)	0.38
ICU visits, no	1 (0‒3)	1 (0‒4)	0.6
**Patients**			
Age, years	68.83 ± 20.8	58.63 ± 14.28	0.13
Female gender, % (no.)	66.7 (4)	37 (17)	0.21
Emergency admission, % (no.)	100 (6)	95.7 (44)	1
APACHE II	31.17 ± 10.13	26.87 ± 7.89	0.24
SAPS II	51.33 ± 28.79	35.91 ± 17.32	0.07
COVID-19, % (no.)	33.3 (2)	39.1 (19)	1
Mechanical ventilation, % (no.)	83.3 (5)	89.1 (41)	0.54
Duration of mechanical ventilation, hours	57.3 (20.7‒212.28)	120.5 (56.18‒304.33)	0.27
ICU length of stay, days	4 (3‒7)	9 (5‒18)	0.1
Died in ICU, % (no.)	33.3 (2)	6.5 (3)	0.1

Significant values are in bold.

Relatives’ age, family satisfaction score, patients’ age, duration of mechanical ventilation, and length of stay expressed as median and interquartile range; IES-R score, Acute Physiology and Chronic Health Evaluation II (APACHE II) score, and Simplified Acute Physiology Score II (SAPS II) expressed as mean and standard deviation.

### Comparisons of characteristics in first and second waves

During the first wave (15.03.2020‒30.04.2020; visits banned), 16 patients/relatives were included in this study. In the second wave (01.10.20‒08.02.21; visits restricted) 36 patients/relatives were included. No patient’s ICU stay covered both waves of the pandemic. Detailed data are given in Table 3. No differences were found with regard to IES-R scores and aFS-ICU 24R scores. The numbers of telephone calls and video calls with the participating relatives were higher in the first wave. No differences between the first and the second wave were observed for the ICU visits made by the participating relatives. The numbers of ICU visits by the patients’ relatives overall were higher during the second wave. Patients’ length of stay in the ICU was longer in the first wave.

**Table 3:** Comparison of characteristics in the first and second waves of the COVID-19 pandemic.

Characteristic	First wave	Second wave	*p* value
**Relatives**			
Age, years	51.25 ± 17.91	47.88 ± 13.51	0.5
Female gender, % (no.)	75 (12)	72.2 (26)	0.84
IES-R score at 3 months	48.69 ± 15.03	48.11 ± 10.59	0.88
IES-R > 33, % (no.)	81.2 (13)	91.7 (33)	0.36
aFS-ICU 24R	37 (37‒40)	40 (37‒40)	0.24
Family member in ICU			
Spouse, % (no.)	43.7 (7)	33.3 (12)	0.51
Parent, % (no.)	12.5 (2)	8.3 (3)	0.64
Sibling, % (no.)	0 (0)	11.1 (4)	0.3
Child, % (no.)	37.5 (6)	47.2 (17)	0.51
Other, % (no.)	6.2 (1)	0 (0)	0.31
ICU diary, % (no.)	56.2 (9)	35.3 (12)	0.2
Previous ICU experience, % (no.)	50 (8)	38.9 (14)	0.55
Care team involved, % (no.)	26.7 (4)	14.3 (5)	0.42
**Telephone calls with nurses, no**	**14 (4.5‒21)**	**4.5 (3‒9)**	**0.01**
**Video calls, no**	**7.5 (3.5‒13)**	**0 (0‒1)**	**< 0.0001**
ICU visits, no	0 (0‒3)	1.5 (1‒5)	0.05
**Patients**			
Age, years	59.19 ± 15.85	60.08 ± 15.23	0.85
Female gender, % (no.)	25 (4)	47.2 (17)	0.22
Emergency admission, % (no.)	100 (16)	94.4 (34)	1
APACHE II	28.93 ± 6.67	26.69 ± 8.95	0.4
SAPS II	39.63 ± 22.87	36.91 ± 17.74	0.65
COVID-19, % (no.)	50 (8)	33.3 (12)	0.16
Mechanical ventilation, % (no.)	93.7 (15)	86.1 (31)	0.65
Duration of mechanical ventilation, hours	133.5 (85.83‒490.93)	108 (31.3‒203.1)	0.09
**ICU length of stay, days**	**18 (7.5‒24.5)**	**5.5 (4‒11.5)**	**0.003**
Died in ICU, % (no.)	18.8 (3)	5.6 (2)	0.16

Significant values are in bold.

Relatives’ age, family satisfaction score, patients’ age, duration of mechanical ventilation, and length of stay expressed as median and interquartile range; IES-R score, Acute Physiology and Chronic Health Evaluation II (APACHE II) score, and Simplified Acute Physiology Score II (SAPS II) expressed as mean and standard deviation.

### Qualitative content analysis

A total of 91 comments on experiences of the participated relatives were divided into three categories: information/communication, emotional support, and technical and emotional aspects of video calls.

Forty-six comments fell into the category information/communication. The relatives experienced good provision of information from the medical staff about the patients’ condition: “I always got information when calling;” “I could always call for information.” However, there were also reports of missing or late information about relevant events: “There was no warning before the intubation.” Only a few comments about shared decision making were recorded: “My father was transferred too early;” “I was glad that my wife was not transferred to another hospital/ICU.” Moreover, the relatives would have preferred having only one contact person for all concerns: “Too many different contacts, for long-term patients one single trusted person would be good.”

Twenty-seven of the 91 comments were assigned to the category emotional support, which included positive memories of emotional support from medical staff, such as listening to the relatives’ worries, responding to their needs, or just being kind. However, it also included burdensome experiences, such as the visiting restrictions; “Quarantine is very difficult”; or less supportive contacts with the medical staff.

In the third category (18 comments), technical and emotional aspects of the video calls, technical difficulties such as poor audio quality were noted: “Very satisfied despite technical problems. Speakers could not be activated but we found a solution together.”

As emotional aspects of the video calls, relatives noted reduced feelings of helplessness and the feeling of greater closeness to the patient after/during the video call: “Seeing the patient creates closeness, it makes things easier.” However, some relatives stated that they were saddened or distressed at the sight of their loved ones in the ICU: “The contact, on the other hand, was also shocking, as the image could be very burdening.” Two relatives noted the need for more flexibility of call options (e.g., member of staff involved, time of day). One relative suggested preparing relatives in advance of the video call for the sight of their critically ill family member (e.g., endotracheal intubation, ECMO, prone position).

## Discussion

Before the COVID-19 pandemic, integration of family members in terms of shared decision making and direct caregiving, e.g., frequent communication and open visiting, helped to improve family-centered care and assisted relatives in coping with post-intensive care syndrome-family (PICS-F)^1,8,10^. During the COVID-19 pandemic, family-centered care has been challenged by measures taken to help prevent the spread of the SARS-CoV-2 virus, such as restricted ICU visiting and social distancing^3–6^. New approaches have been proposed to meet the needs of patients’ relatives during restriction of ICU visits^5,13^. Video calls have been recommended to maintain communication between relatives and ICU staff^15,16,26,27^.

In a prospective single-center mixed-method study, we investigated the effect of video calls versus standard care on PTSD symptoms in the relatives of ICU patients during ICU visiting restrictions in the course of the COVID-19 pandemic. We found that video calls as an element of family-centered care had no significant effect on PTSD symptoms in these relatives.

There are several possible reasons why the IES-R scores remained high in the video call group of relatives. First, shared decision making appears to be more difficult in video calls than in face-to-face communication^16,26^. However, compared to the standard care without video calls, shared decision making remains difficult. Unfortunately, the impact of a compromised decision making process during suspension or restriction of ICU visits remains unclear in this study. Further studies are needed.

Second, direct caregiving to the family member is not possible during restricted ICU visiting, and relatives may be burdened by strong feelings of powerlessness^3^. Moreover, a no-visiting policy appears to impose an exceptional load on relatives and may cause feelings of “stolen moments”^3^ and “lost contact with loved ones”^7^. This may not be prevented by video calls. Therefore, restricted ICU visiting, together with a general burden during the COVID-19 pandemic^28^ may explain why the IES-R scores remain higher than before the pandemic^8^, with a large proportion of relatives experiencing severe PTSD symptoms^7^.

We observed no significant differences in family satisfaction with care between the VCG and the SCG. The scores were high for both groups, which may appear to imply that, despite restriction or suspension of ICU visiting, family satisfaction with support, communication, information, and the decision-making process remains satisfactory. In addition, conventional telephone calls may be sufficient to meet these types of needs for family members. Even though family satisfaction with care was high, PTSD symptoms were high for both the VCG and the SCG relatives^7,29^.

The PTSD symptoms remained high in relatives during the second wave, when visiting was less restricted. It could be that open visitation polices do not have a direct impact have on relatives’ PTSD symptoms. However, it is difficult to compare 1 h a day of visitation to completely open visitation, so more research is needed on this.

Most of the video calls were made during the first wave of the COVID-19 pandemic, when only end-of-life visits to the ICU were permitted. All members of the SCG participated during the second wave, when visiting was allowed but restricted (to one relative per day for 1 h). Interestingly, PTSD symptoms or family satisfaction may not be changed by suspension or restriction of visits in combination with conventional telephone calls and video calls. It is tempting to speculate that video and conventional telephone calls are a suitable substitute for ICU visits. However, open visiting policies may affect family satisfaction positively^30^, although the impact on PTSD symptoms remains unclear^30–32^. Although on the one hand some families shared that they had a reduced feeling of helplessness and a feeling of closeness to the patient during the video calls, on the other hand some reported feelings of sadness and distress caused by seeing their loved ones in the ICU during the video calls. This potential negative effect of video calls should be borne in mind when considering their introduction. The use of checklists for video calls may help ICU staff to prepare relatives for the sight of the patient (e.g., ECMO, endotracheal intubation, prone position). However, clinical data on the effect of checklists are sparse, so more research is needed on their effectiveness in this situation^15,16^. Other research has suggested that imposition of ICU visiting restrictions during the COVID-19 pandemic may have led to an extraordinary burden on relatives^3^. With this in mind, together with the fact that several important elements of family-centered care are not feasible during restricted ICU visits, it is worth considering revision of the ICU visiting policy. Indeed, resumption and extension of ICU visits may be required to meet the needs of patients’ relatives during the ongoing COVID-19 pandemic^1,2^. Furthermore, the poor evidence that single interventions such as video calls reduce PTSD symptoms in relatives underlines the need for a multifaceted approach in the sense of family-centered care^12,32^.

Gender, COVID positivity, disease severity, and length of stay may impact on family satisfaction. Notably, the adapted version of the FS-ICU 24R used in our study focuses on information provision, emotional support, communication, and the shared decision-making process, which are likely to be independent from these data. In contrast, there is some evidence that female gender is related to PTSD symptoms in relatives^8^. In the pandemic, as earlier, female gender and ARDS in patients with COVID-19 are associated with increased PTSD symptoms on the part of relatives^33^. However, in the COVID-19 pandemic it seems that general disease severity and length of stay have no impact on severe PTSD symptoms in relatives^7^. Potential effects on relatives’ quarantine or isolation could theoretically influence the occurrence or severity of PTSD symptoms. However, the data on this issue remain controversial^28,34,35^. Furthermore, data on social support additional to the care-team support provided were missing, and this factor could also affect PTSD symptoms^36–38^.

This study features several important limitations. First, the single-center design reduces the generalizability of the findings. The strengths of the study, on the other hand, are the mixed-method approach, the prospective design, and the power calculation for sample size to estimate differences in PTSD symptoms.

With regard to the specific sample size calculation only for the effect of video calls to PTSD symptoms, other findings should be interpreted with caution. The use of a convenience sample strategy should be considered in the interpretation of the data. To comply with family-centered care in the COVID-19 pandemic, we refrained from using a randomized controlled design so that no relatives would be denied video calls. However, randomized controlled trials are needed to generate more evidence. Finally, no information was available on pre-existing PTSD or psychological disorders in the participating relatives.

## Conclusions

The use of video calls appears to have little effect on reducing PTSD symptoms in patients’ relatives at times of suspension or restriction of visits to the ICU, as in the COVID-19 pandemic. Family satisfaction may be better when visiting is restricted than when it is banned entirely. Further research is required to gain more knowledge about the factors involved in the development of PTSD symptoms in relatives of ICU patients during suspension or restriction of ICU visits. In the future, multifaceted strategies should be developed to support relatives of ICU patients in such situations.

## Data Availability

All data generated and/or analyzed during this study are included in this published article.

## Abbreviations

PTSD: Post-traumatic stress disorder
ICU: Intensive care unit
IES-R: Impact of Event Scale-Revised
IQR: Interquartile range
aFS-ICU 24R: Adapted Family Satisfaction in the Intensive Care Unit 24-Item-Revised questionnaire
SD: Standard deviation
APACHE II: Acute Physiology and Chronic Health Evaluation II
SAPS II: Simplified Acute Physiology Score
VCG: Video call group
SCG: Standard care group
PICS-F: Post-intensive care syndrome-family
ECMO: Extracorporeal membrane oxygenation
